# Genomic clonal evolution correlated with phenotype and prognosis in gastric cancer

**DOI:** 10.1002/ctm2.799

**Published:** 2022-04-05

**Authors:** Jie Ge, Xuan Li, Zhenghao Deng, Xuan Gao, Yaoyao liu, Xingui Xiong, Xianhui Zhao, Huan Peng, Xin Yi, Xuefeng Xia, Zihua Chen, Lifeng Li, Haiyan Zhou, Heli Liu

**Affiliations:** ^1^ Department of Gastrointestinal Surgery Xiangya Hospital Central South University Changsha China; ^2^ Department of Pathology Xiangya Hospital Central South University Changsha China; ^3^ Institute of Combined Traditional Chinese and Western Medicine Xiangya Hospital Central South University Changsha China; ^4^ The Hunan Provincial Key Laboratory of Precision Diagnosis and Treatment for Gastrointestinal Tumor Changsha China; ^5^ International Joint Research Center of Minimally Invasive Endoscopic Technology Equipment and Standardization Changsha China; ^6^ Geneplus‐Beijing Beijing China; ^7^ State Key Laboratory of Microbial Resources Institute of Microbiology Chinese Academy of Sciences Beijing China; ^8^ GenePlus‐Shenzhen Clinical Laboratory ShenZhen China

Dear Editor,

Gastric cancer (GC) is a highly heterogeneous disease with a dismal prognosis at both phenotypical and molecular levels.^1^ The Lauren type is currently the most useful and widely used in GC.^2^ However, the prognosis and molecular characteristics of Lauren type have not been fully described. Additionally, although several molecular classifications have been proposed,^3^, ^4^ clinically‐relevant subtypes are still urgently needed. Here, we systematically investigated the molecular landscape and evolution features of 169 primary GC samples among Lauren type. We identified a prognostic‐relevant subtype based on clone number (CN). Patients with high CN showed high tumour mutation burden (TMB) and significantly enriched in Adherens junction, ERBB2 regulates cell motility, and signaling by WNT pathway, indicating CN may have potential risk of tumour metastasis and benefit from immunotherapy. Our findings may inform the exploration of patient stratification and personalized therapy, as well as new clinical trials designed for the selection of combination therapy strategies.

We enrolled 169 patients with formalin‐fixed paraffin‐embedded samples of matched tumour and adjacent tissue, including extended Lauren type: intestinal type (IT, *n* = 26), mixed type (MT, *n* = 35), non‐signet ring diffuse type (NSRD, *n* = 77), and signet ring type (SRT, *n* = 31) with signet ring cells accounting for more than 10%.^5^ The median sequencing depth was 747× (range, 373–1186×) for tumour and was 428× (range, 221–960×) for adjacent tissue (Figure S1). We obtained 2847 Single Nucleotide Variations (SNVs) and 44 indels. The median TMB was 10.56 per Mb (mean 16.43 per Mb). The most recurrent mutant genes included *TP53* (39.1%), *CDH1* (28.4%), *ARID1A* (24.9%), *TTN* (24.3%), and *MUC16* (19.5%), which was consistent with previous studies (The Cancer Genome Atlas (TCGA): 48.1%, 8.4%, 25.8%, 54.9%, 33.9%; ACRG: 40%, 4%, 17.8%, 38.2%, 25.8%; oncosg: 47.6%, 9.5%, 13.6%, 38.1%, 19.7%; respectively. Figure S2A).^3^, ^4^, ^6^ *MUC16*‐ and *TTN*‐mutated samples showed higher TMB value (Figure S2B,C,E). Only Adenomatous Polyposis Coli (*APC*) (*p* = .038) showed significant difference (Figure 1, Figures S3–S4), suggesting high‐frequency mutated genes have similar variation pattern in the four types. There was no significant difference in sex, age, venous invasion, perineural invasion, treatment regimen, TNM stage, tumour site, tumour size, and MSI status except CEA(*p* = .0221) and CA199 index (*p* = .03) (Table S1, Figure S5C,D). Patients with high tumour markers generally have worse prognosis (Figure S5A,B). However, we found only patients in NSRD with CEA‐high or CA199‐high had significantly shorter outcomes (Figure S5E,F), suggesting the importance of stratified management for patient care. The mutational signature analysis also showed only signature 3 and signature 17 were not enriched in NSRD and SRT, respectively (Figure 1, Figure S6).

**FIGURE 1:**
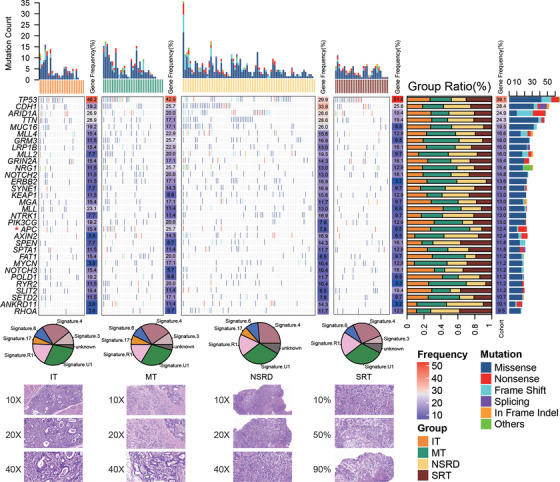
Comparison of molecular landscape in four histological types. The genes with a mutation frequency greater than 10% were shown in heatmap. Each column represents a single sample. All 169 samples were divided into four groups according to the expanded Lauren type and were presented separately. The numbers next to the heatmap represent mutation frequency. The percentage stack histogram on the right shows the proportion of each group. The rightmost bar represents the variation type of gene. The upper bar represents mutation number. The bottom pie chart shows the proportion of mutation signature in the four types. Representative immunohistochemistry of Lauren type is displayed below the pie chart at different resolutions of 10×, 20× and 40×, respectively. The immunohistochemistry of the signet ring cells, which account for 10%, 50% and 90%, is also shown respectively

In terms of comprehensive indicators, we compared the TMB score, mutant‐allele tumour heterogeneity (MATH) score,^7^ and variant allele frequency (VAF) from three dimensions: mutational burden, mutation heterogeneity, and allelic mutation frequency. We found no significant difference in TMB and MATH values between the four types except VAF (Figure 2A–C), suggesting VAF may influence histological types. We then preformed pyclone analysis.^8^ We defined the highest cell prevalence cluster as clone and the other as subclone. Pyclone inferred 379 clones (IT, 59, 13.9%; MT, 70, 9.9%; NSRD, 176, 12.9%; SRT, 74, 18.8%) and 2514 subclones (IT, 366, 86.1%; MT, 640, 90.1%; NSRD, 1188, 87.1%; SRT, 320, 81.2%). The median CN was 7 (range, 1–54). Highly specific clonal genes were observed in each type (unique ratio: IT, 30.5%; MT, 22.9%; NSRD, 32.9%; SRT, 28.4%) (Figure 2D), and subclonal gene has similar phenomenon (Figure 2E). The results indicated the differences of gene mutation process among the four types.

**FIGURE 2:**
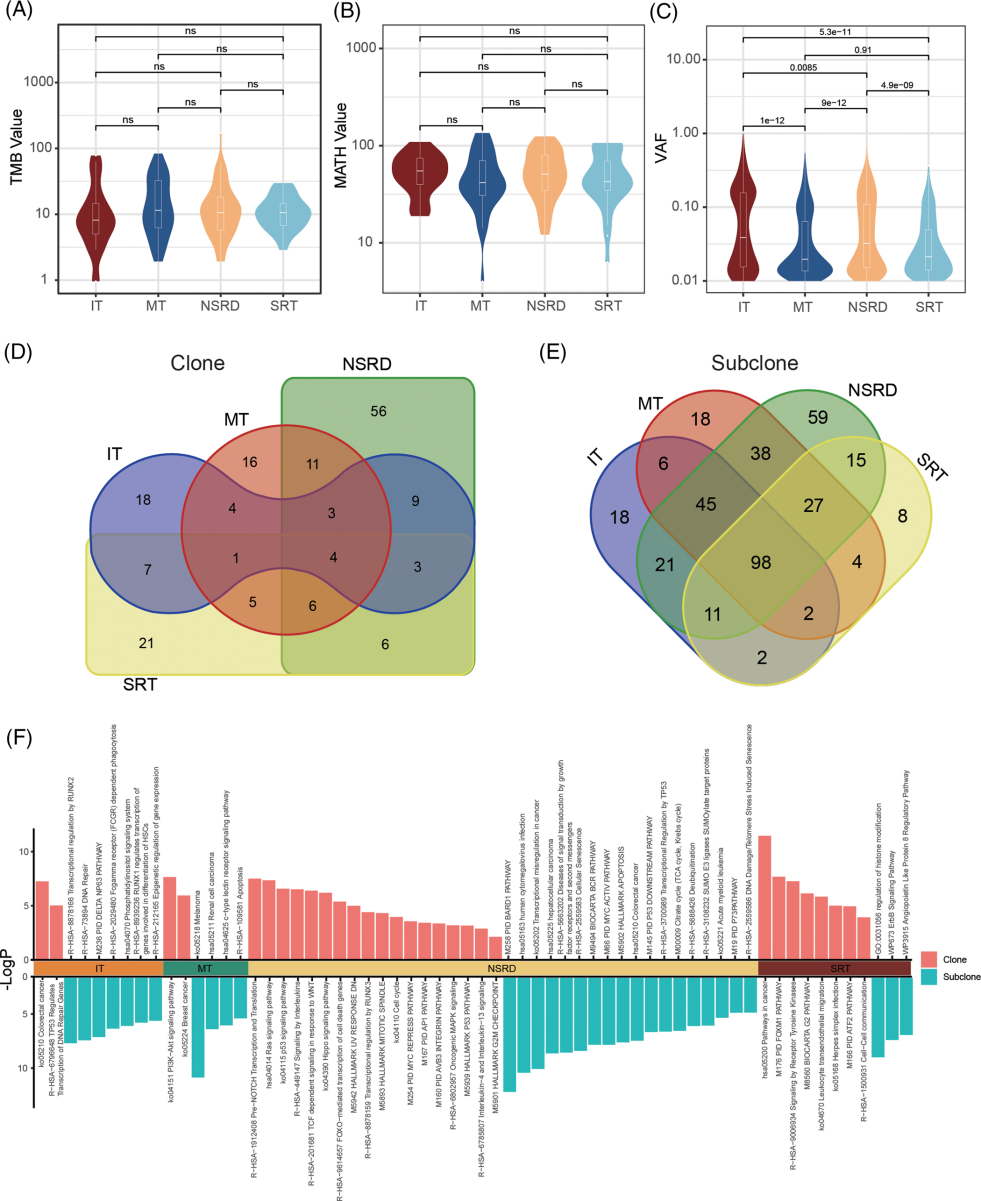
Clonal evolution analysis of four histological types. Violin plot shows the comparison of tumour mutation burden (TMB) (A), mutant‐allele tumour heterogeneity (MATH) (B) and variant allele frequency (VAF) (C), respectively. The ns above horizontal line indicates the *p* value of Mann–Whitney *U* test greater than .05, and the number above horizontal line indicates *p* value. Venn diagrams show the sharing gene number of clone (D) and subclone (E) in four types. (F) Clonal and subclonal gene enrichment pathways in four types

To clarify the functional role of clonal and subclonal genes, we performed pathway enrichment analysis. The result showed clonal genes in IT were significantly enriched in TP53 regulates transcription of DNA repair genes pathway, and subclonal genes were in DNA repair pathways. We also found clonal and subclonal genes in MT were enriched in the PI3K‐Akt signaling pathway and c‐type lectin receptor signaling pathway, respectively. Several classical tumour‐related pathways were enriched in the NSRD, such as pre‐NOTCH transcription and translation, Ras signaling pathway, and p53 signaling pathway. Additionally, signaling by receptor tyrosine kinases and ErbB signaling were significantly enriched in the SRT, respectively (Figure 2F). The different functional enrichment features among the four types suggest the clonal evolution patterns may be related to histological phenotypes.

To evaluate the clonal heterogeneity process, the distribution of high‐frequently mutant genes was tested. We found that the same gene can be either a clone gene or a subclone gene in different samples, indicating gene clonal heterogeneity (Figure 3A). Furthermore, CN was significantly correlated with the maximum VAF (Figure 3B). To further illustrate the clinical relevance of CN, we observed the characteristics distribution of CN with the overall survival data (Figure 3C) and patients with CN ≥ 7 was associated with poor outcomes (Figure 3D). Besides, CN stratification remained significantly associated with survival in the multivariate setting of Cox model (Figure 4A). We further excluded the interference of phenotype and stage on CN stratification by statistical test (Figure 4B,C). CN may be an independent prognostic factor in GC.

**FIGURE 3:**
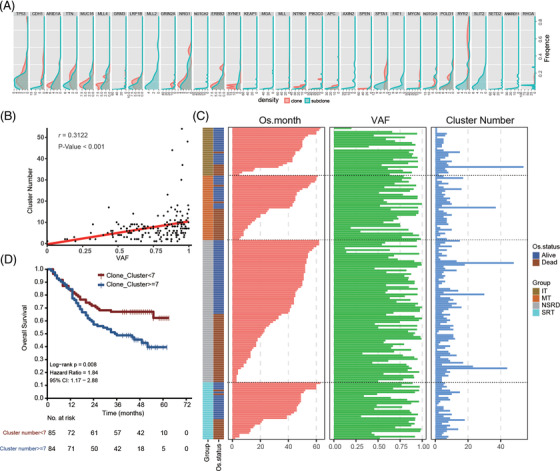
Correlation analysis of clone number stratification and prognosis. (A) The density distribution of the top 10% mutant genes with clonal and subclonal attribute. (B) Correlation analysis between clone number and variant allele frequency (VAF). (C) Comprehensive display of overall survival, VAF and clone number distribution. (D) The prognosis of clonal number stratification

**FIGURE 4:**
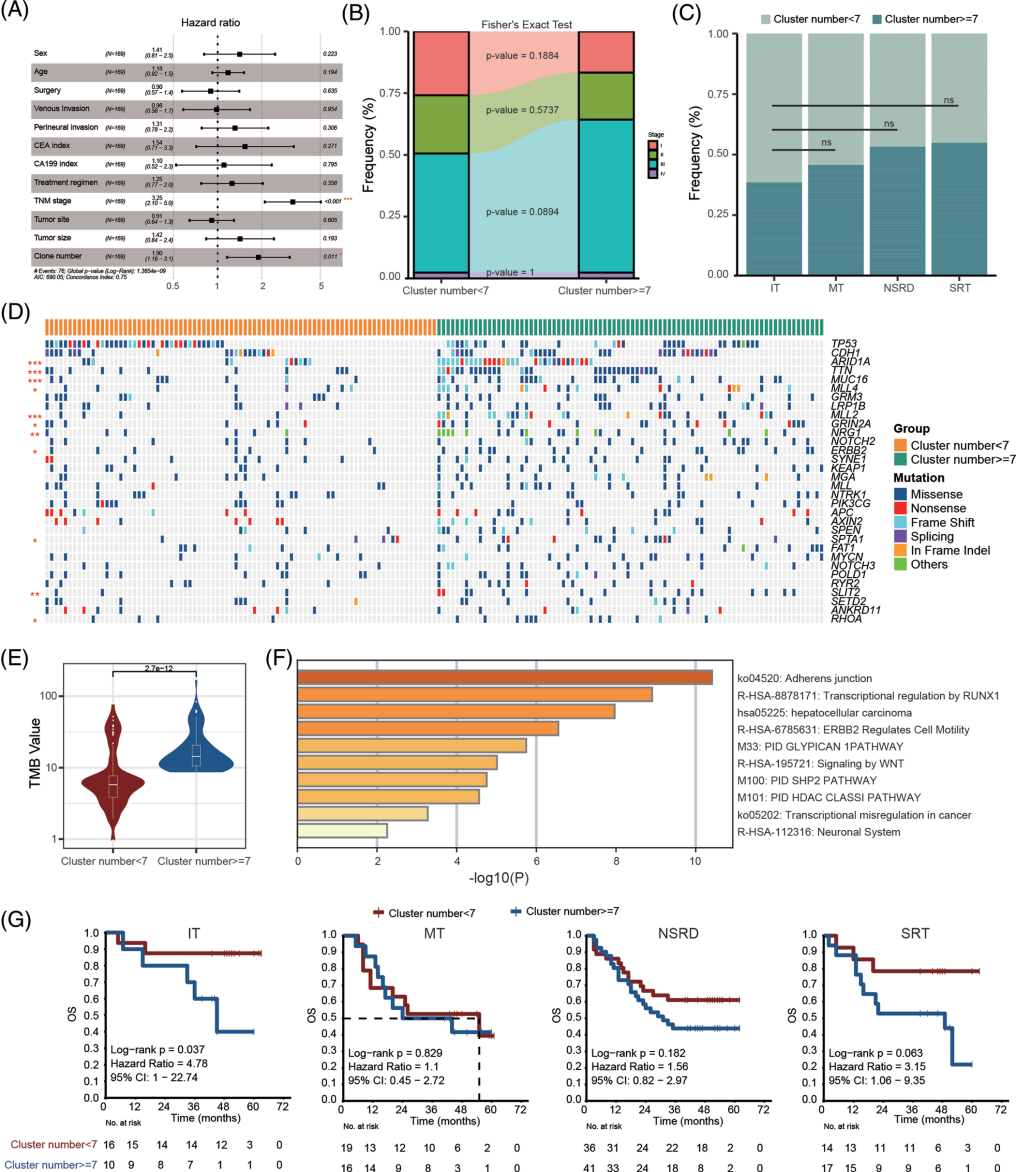
Comparative analysis of the clone number stratification. (A) Multivariate Cox hazard analysis of clinical information and clone number stratification. (B) The proportion of pathological stage in the clone number stratification with Fisher's exact test. (C) The distribution of clone number stratification in the four types, ns means Fisher's exact test *p* value greater than .05. (D) Comparison of high‐frequency mutant genes in clone number stratified. Each column represents a single sample. Different color indicates mutation types. The red asterisk on the left indicates the *p* value interval (*, [.05, .01]; **, [.01, .001]; ***, [.001, 0]). (E) The violin plot shows the tumour mutation burden (TMB) value of clone number stratification. (F) Enrichment of differential gene pathways based on clone number stratification. (G) Prognostic analysis of clone number stratification in four histological types

Detailed mutational landscape analysis of CN stratification showed the mutation frequencies of *ARID1A*, *TTN*, *MUC16*, *MLL4*, *MLL2*, *GRIN2A*, *NRG1*, *ERBB2*, *SPTA1*, *SLIT2*, and *RHOA* genes were significantly higher in CN‐high group (Figure 4D). Higher TMB was also observed in CN‐high group (Figure 4E). Patients with high CN may benefit from immunotherapy. Functional enrichment analysis showed that Adherens junction, ERBB2 regulates cell motility, and signaling by WNT were significantly enriched in CN‐high group (Figure 4F), indicating patients with high CN may have a high risk of metastasis. We also found the prognosis of IT CN ≥ 7 subgroup was significantly worse than that of CN < 7 subgroup, and consistent trend was observed in NSRD and SRT (Figure 4G). Thus, combining CN and histological phenotype may be an actionable marker for clinical prognosis stratification.

In summary, we highlight the molecular, evolutionary and prognostic heterogeneity of GC phenotype at multi‐dimensionally levels. The clonal evolution patterns of the four histological types showed different characteristics, and CN may be a molecular classification indicator for patient stratification.

## CONFLICT OF INTEREST

The authors declare there is no conflict of interest.

## Supporting information

SUPPORTING INFORMATION

SUPPORTING INFORMATION

SUPPORTING INFORMATION

SUPPORTING INFORMATION

SUPPORTING INFORMATION

SUPPORTING INFORMATION

SUPPORTING INFORMATION

SUPPORTING INFORMATION
