# Supplementary figures and images for: Genomic clonal evolution correlated with phenotype and prognosis in gastric cancer
Source: Clin Transl Med. 2022 Apr 5;12(4):e799. doi: 10.1002/ctm2.799 (PMC8982317; doi:10.1002/ctm2.799)

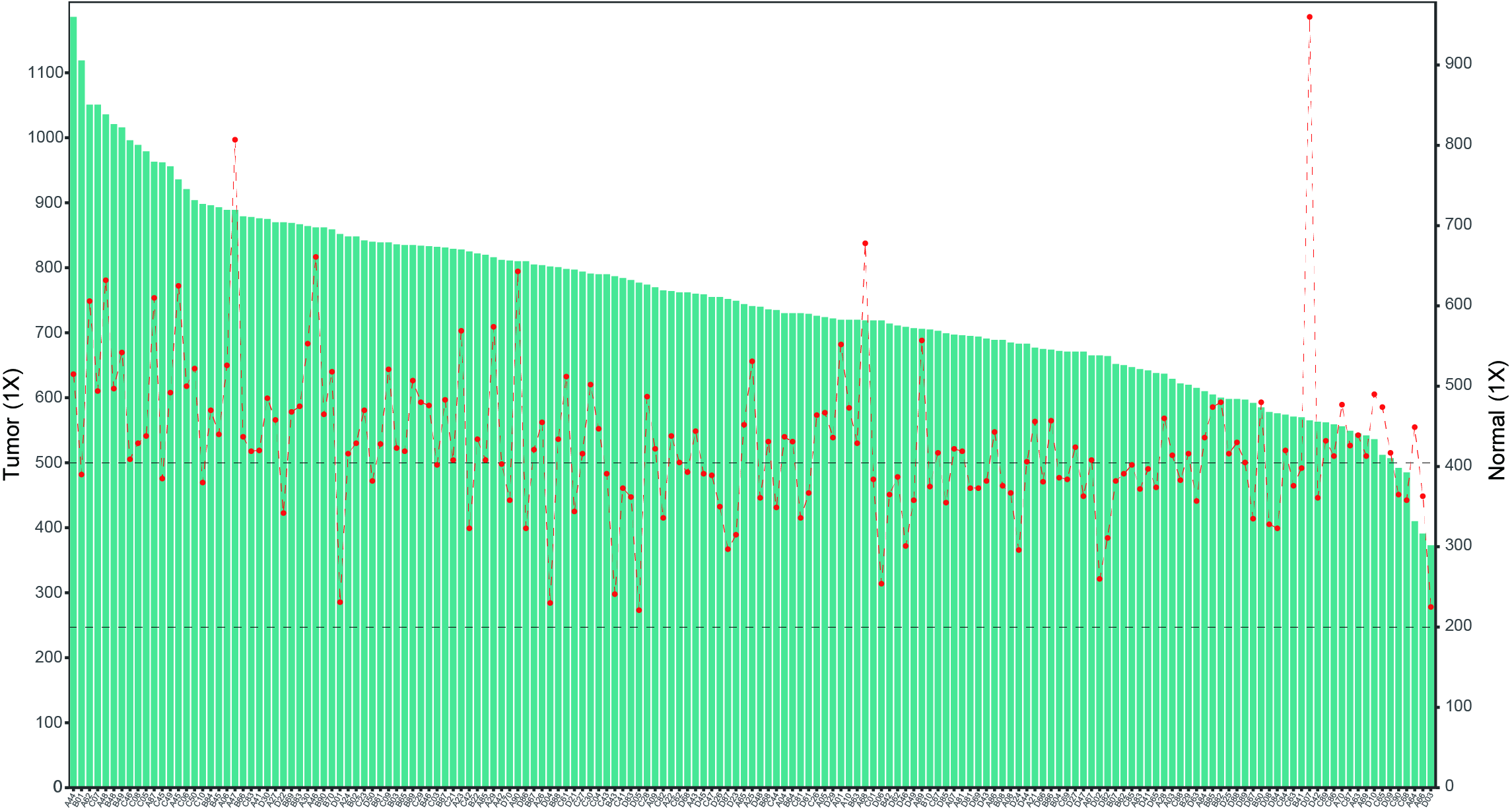

Supplement: Supplementary file 1 — SUPPORTING INFORMATION [file CTM2-12-e799-s001.tif]

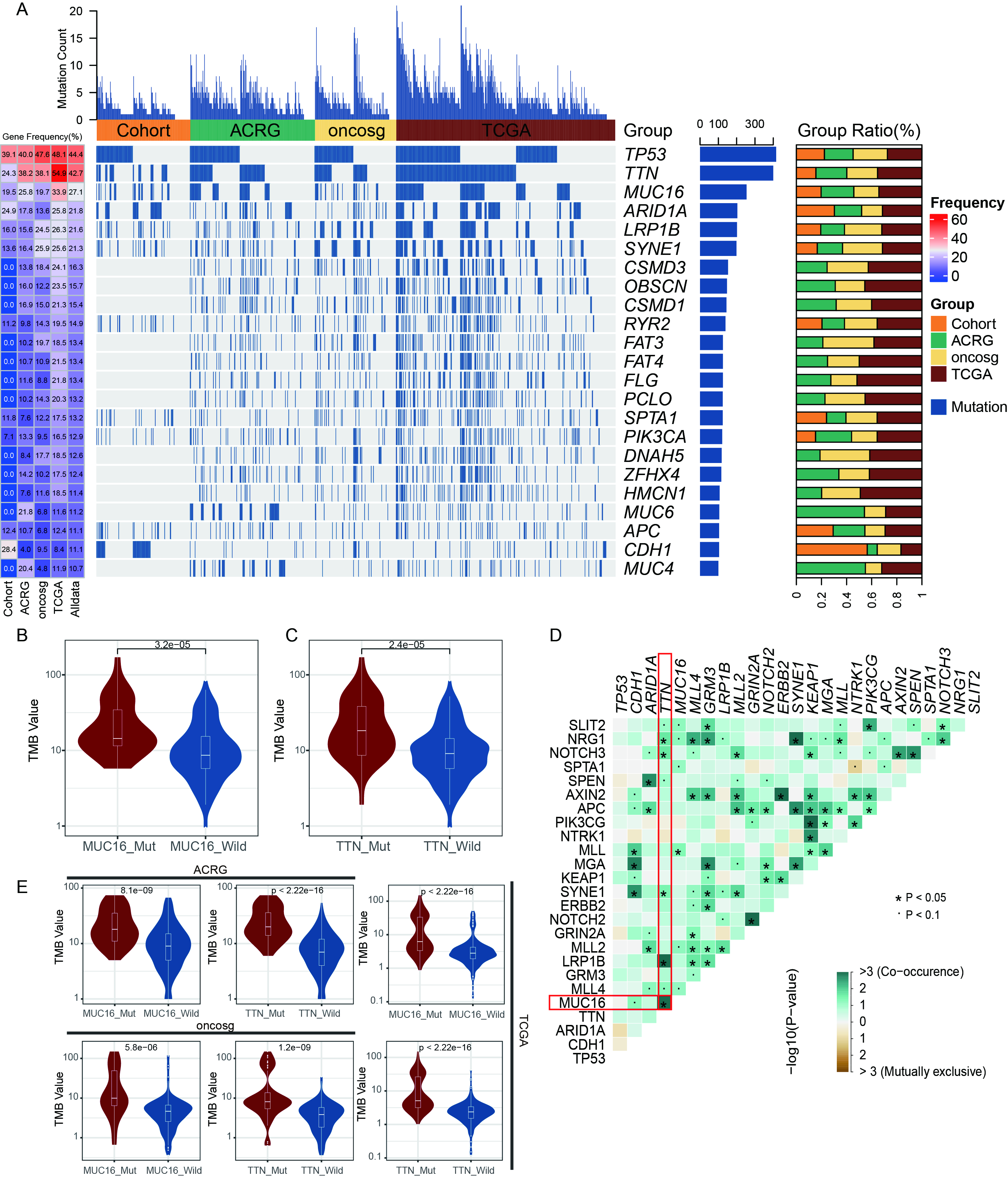

Supplement: Supplementary file 2 — SUPPORTING INFORMATION [file CTM2-12-e799-s007.tif]

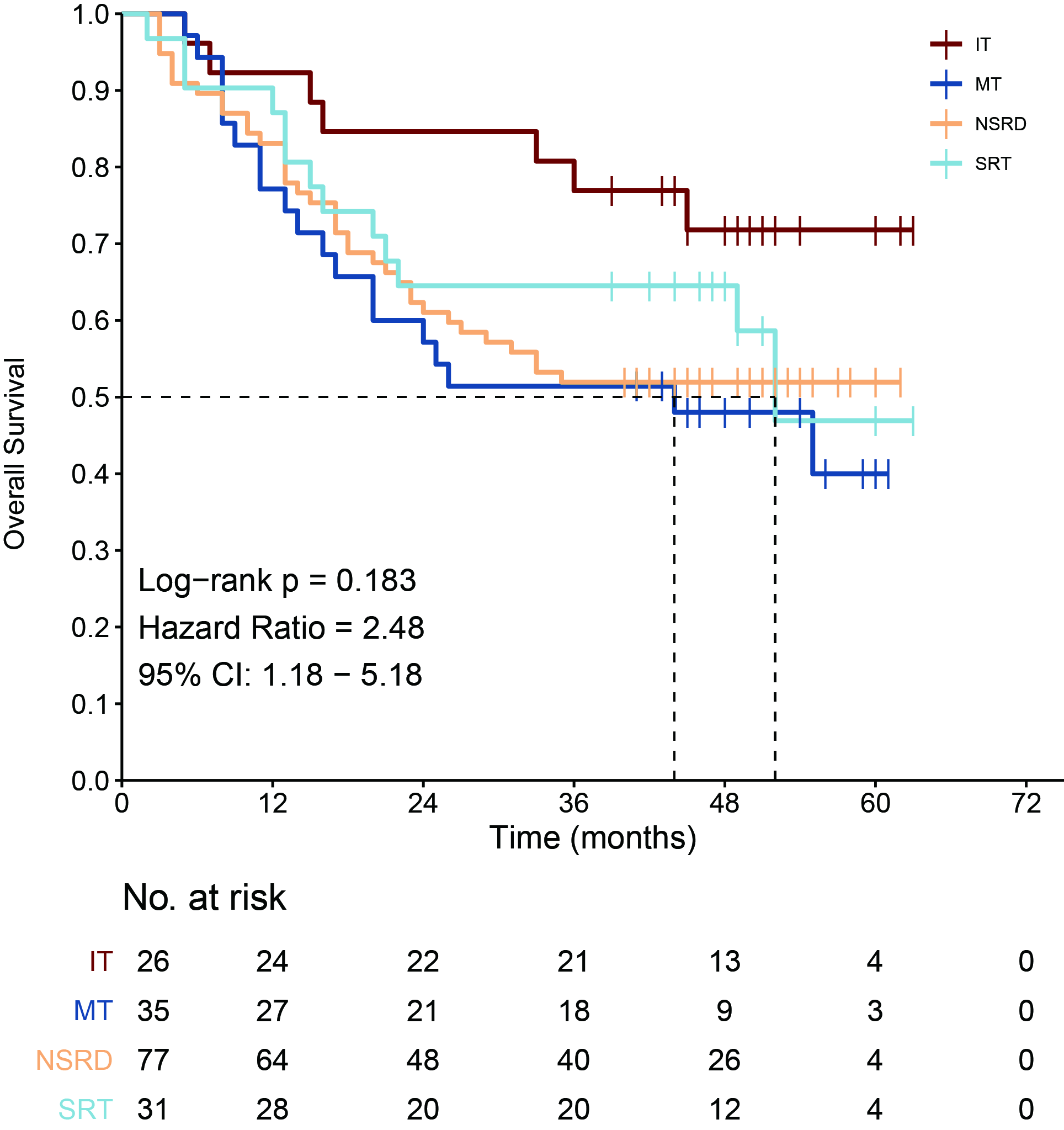

Supplement: Supplementary file 3 — SUPPORTING INFORMATION [file CTM2-12-e799-s008.tif]

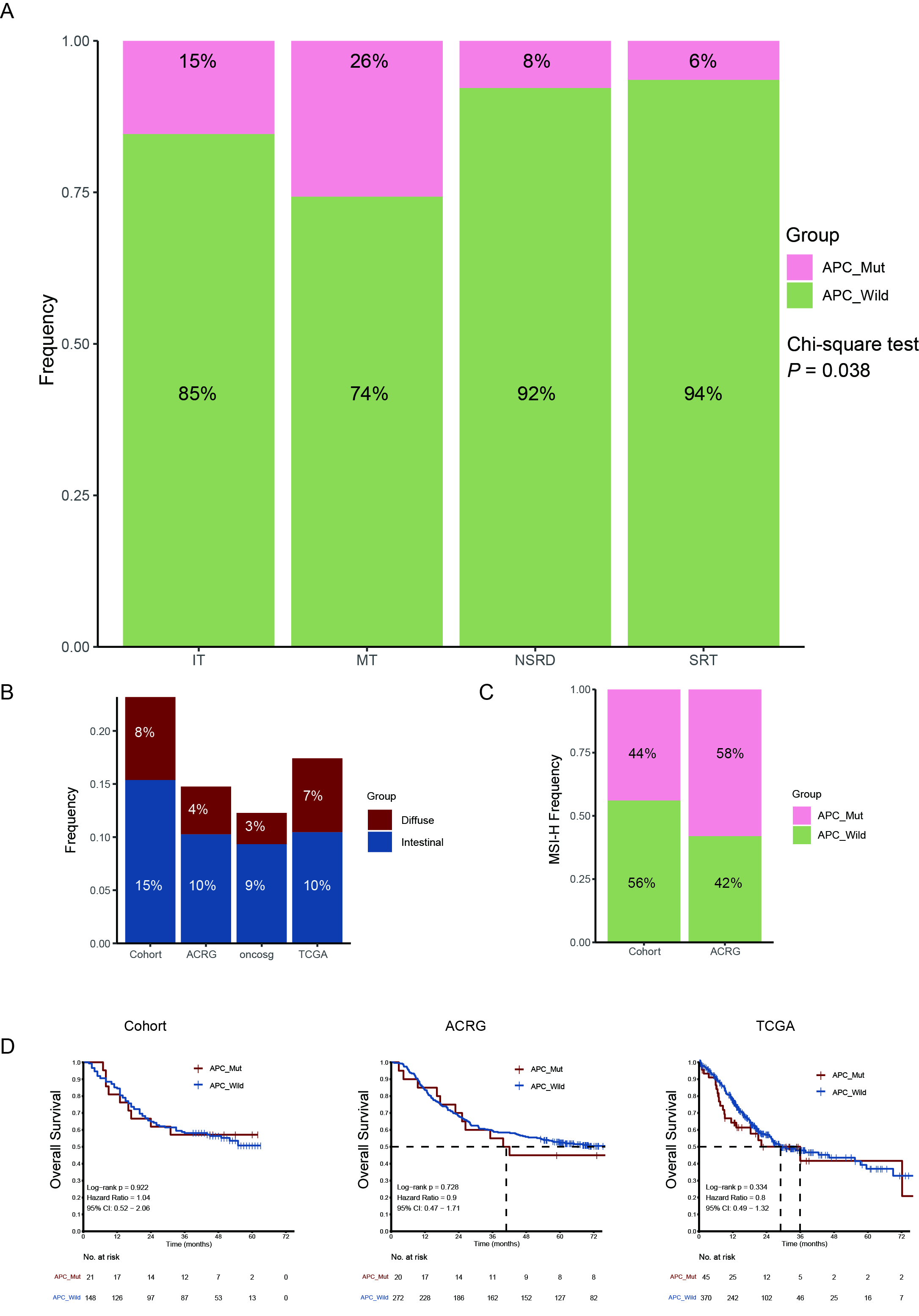

Supplement: Supplementary file 4 — SUPPORTING INFORMATION [file CTM2-12-e799-s002.tif]

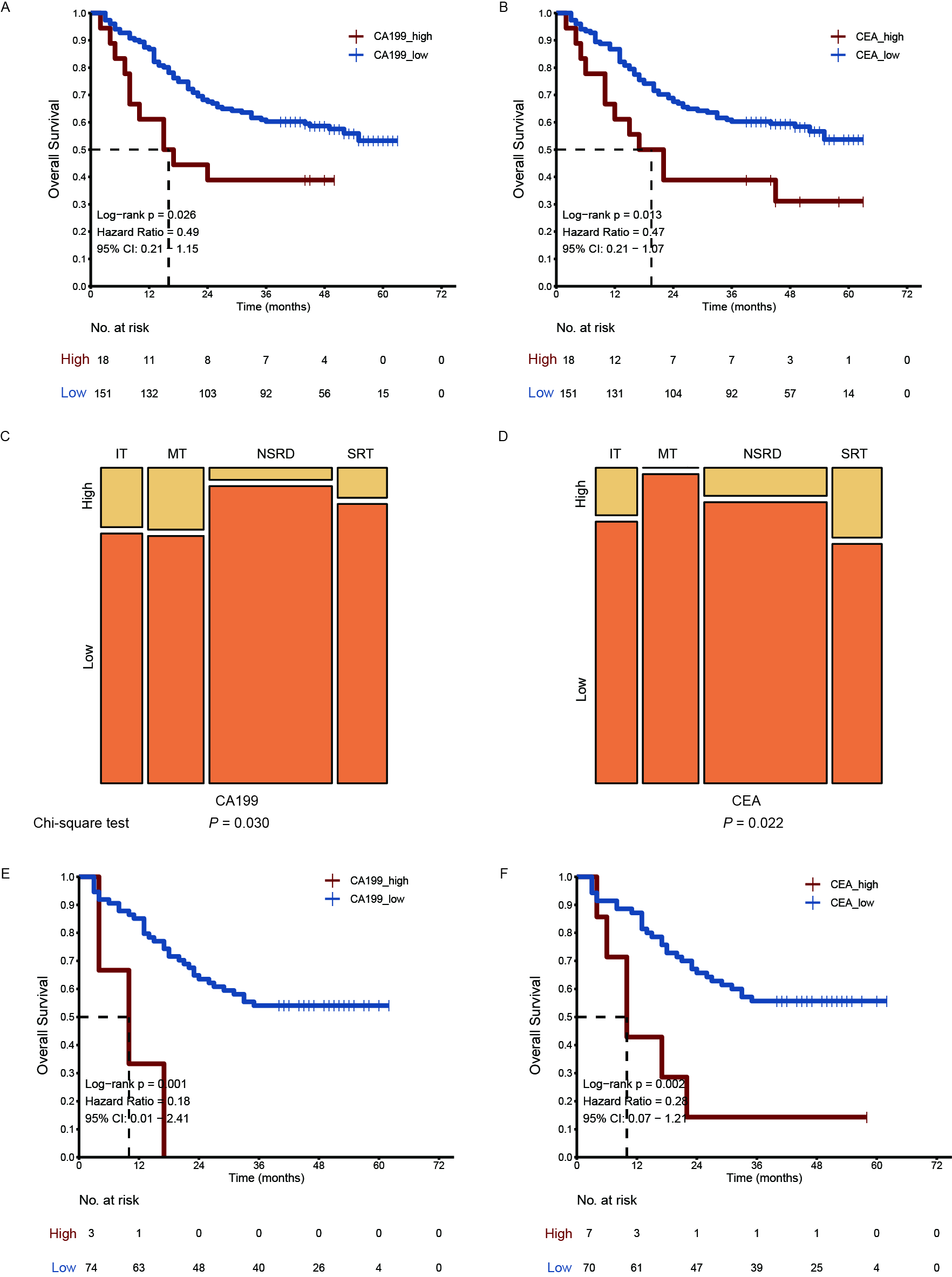

Supplement: Supplementary file 5 — SUPPORTING INFORMATION [file CTM2-12-e799-s004.tif]

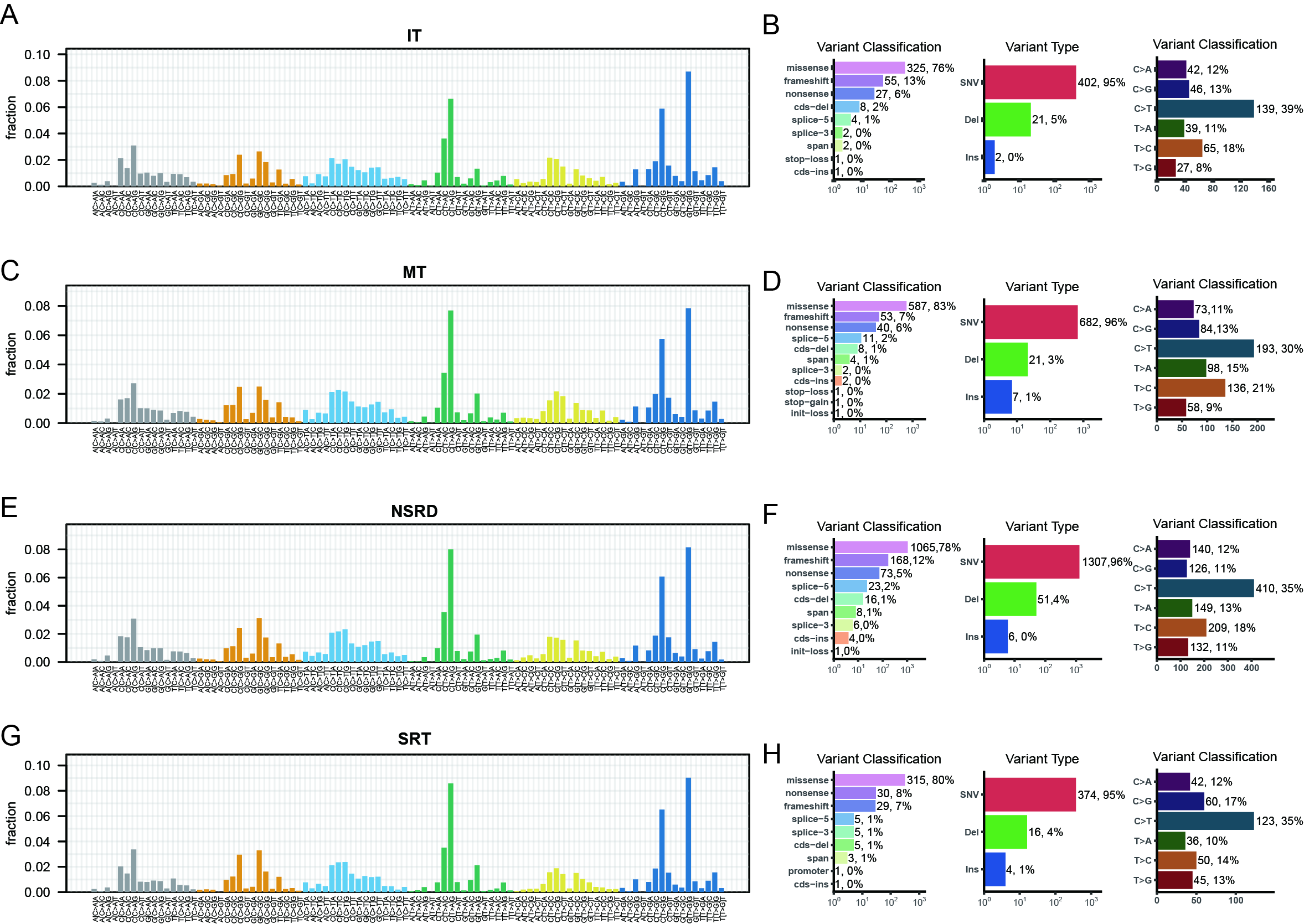

Supplement: Supplementary file 6 — SUPPORTING INFORMATION [file CTM2-12-e799-s003.tif]
