# Supplementary material for: Genomic clonal evolution correlated with phenotype and prognosis in gastric cancer
Source: Clin Transl Med. 2022 Apr 5;12(4):e799. doi: 10.1002/ctm2.799 (PMC8982317; doi:10.1002/ctm2.799)
Supplement: Supplementary file 7 — SUPPORTING INFORMATION [file CTM2-12-e799-s005.docx]

**Supplementary information**

**Materials and Methods**

**Clinical cohort**

169 patients diagnosed with GC at Xiangya Hospital, Central South University, from January 2015 to December 2017 were enrolled in this retrospective cohort study. The clinical characteristics of all patients were summarized in table S1. This study was approved by the Medical Ethical Committee of Xiangya Hospital. All participants provided informed written consent before undergoing any study-related procedures. This study was performed under the Declaration of Helsinki.

**Sample collection and DNA extraction**

Tumor tissues were sampled via surgery. Formalin fixation and paraffin embedding were then performed, followed by histologic section preparation. Genomic DNA was isolated from FFPE tumor samples using the QIAamp DNA FFPE Tissue Kit (Qiagen GmbH, Hilden, Germany), according to the manufacturer's protocol. The DNA concentration was measured using the Qubit dsDNA HS (High Sensitivity) assay kit in the Qubit fluorometer (Invitrogen; Thermo Fisher Scientific, Inc., Waltham, MA, USA). To test the DNA integrity, 200 ng extracted DNA was loaded onto the 1% agarose gel with λ-Hind III digest DNA marker (Takara Biotechnology Co., Ltd., Dalian, China). The DNA samples that were longer than the second largest bonds (9,416 bp) of λ-Hind III digest DNA marker were considered integrated samples and used for subsequent analysis.

**Library preparation**

Tumor DNA was sheared into 200-250-bp fragments using a Covaris S2 instrument (Woburn, MA, USA), and indexed NGS libraries were prepared using the DNA Library Preparation Kit for MGISeq-2000 (BGI, Shenzhen, China). Additional detailed information regarding library preparation was described by Lv et al(1).

**Target region capture and next-generation sequencing**

All libraries were hybridized to custom-designed biotinylated oligonucleotide probes (IDT, Coralville, IA, USA) covering 382 genes. All included genes are shown in table S1. DNA sequencing was performed using the MGISeq-2000 Sequencing System (BGI, Shenzhen, China) per the manufacturer’s guideline, which generated 3 Gb of data from tumor DNA. Additional detailed information regarding target region capture and NGS was described by Lv et al(1).

**Raw data processing**

After removing raw reads containing adaptor sequences, those with more than 50% low-quality base, reads, or more than 50% N bases reads were mapped to the reference human genome (GRCh37) using the Burrows-Wheel Aligner (<http://bio-bwa.sourceforge.net/>) with default parameters. Duplicate reads were identified and marked with Picard’s Mark Duplicates tool (<https://software.broadinstitute.org/gatk/documentation/tooldocs/4.0.3.0/picard_sam_markduplicates_MarkDuplicates.php>) for tumor DNA data. Errors introduced by PCR or sequencing were corrected according to clustered reads. Local realignment and base quality recalibration were performed using The Gene Analysis Toolkit (<https://www.broadinstitute.org/gatk/>).

**Somatic mutation calling of tumor DNA**

Somatic single-nucleotide variations (SNVs) were called using the MuTect2 algorithm (<https://software.broadinstitute.org/gatk/documentation/tooldocs/3.8-0/org_broadinstitute_gatk_tools_walkers_cancer_m2_MuTect2.php>). Candidate mutations were filtered if 1) The allele frequency was less than 1%; 2)Variants were filtered as cross-contamination if present in >0.1% samples in single nucleotide polymorphism (SNP) databases (dbsnp, <https://www.ncbi.nlm.nih.gov/projects/SNP/>; 1000G, <https://www.1000genomes.org/>; ESP6500, <https://evs.gs.washington.edu/>; ExAC, <http://exac.broadinstitute.org/> ). 3) The SIFT score >0.05 or PolyPhen2 score <0.85 but keep harmful mutations that can cause disease. The final candidate variants were all manually verified in the Integrative Genomics Viewer (IGV), and the remaining mutations were considered validated somatic variants(2).

**PyClone analysis**

PyClone was used to analyze the clonal population structure of tumor samples from each patient(3). PyClone infers the clonal composition by grouping single nucleotide variation (SNV) with similar cell-frequencies together. Variants located in the cluster with the mean cancer cell fraction (CCF) were defined as clonal and subclone.

**MutSigCV analysis**

MutSigCV(4) software was used to identify significantly mutated genes. MutSigCV uses the estimated background mutation rate of silent mutations to quantify the significance of non-silent mutations in genes, with other confounding covariates taken into account.

**Pathway enrichment analysis**

The online database metascape (http://metascape.org) was used to conduct pathway and process enrichment analysis(5). n out study, the Gene Ontology (GO) terms for biological process, Kyoto Encyclopedia of Genes and Genomes (KEGG) pathways, Reactome Gene Sets, Canonical Pathways and PANTHER Pathway were enriched based on the Metascape online tool. Only terms with P-value < 0.01, minimum count of 3, and enrichment factor of >1.5 were considered as significant.

**Mutation signature analysis**

We delineated mutation signatures using the computational framework proposed by Alexandrov and colleagues(6). The mutation signature takes into account the six types of base substitutions, namely C>A, C>G, C>T, T>A, T>C, T>G, and the 5' and 3' base, and there are 96 possible combinations of mutations to characterize base substitution information. This 96 substitution classification is particularly useful for distinguishing mutational signatures which cause the same substitutions but in different sequence contexts.

**MATH determination**

The MATH value of each allele was calculated from the median absolute deviation (MAD) and the median of its mutant AFs: MATH = 148.26 × MAD/median. The key purpose of the MATH value is to reflect the fluctuation range of AFs in the same sample and can be used as a measure of genomic heterogeneity(7).

**Statistical Analysis**

The χ2 test or Fisher’s exact test was used to compare categorical variables and the nonparametric test for continuous variables. The Kaplan-Meier method with the log-rank test was used to calculate the probability of OS. The effect of risk factors on OS was evaluated by the Cox proportional hazards regression model. All statistical analyses and presentations were performed using R v4.0.5. Statistical significance was set at p < 0.05.

**Reference**

1. Lv X*, et al.* (2017) Detection of Rare Mutations in CtDNA Using Next Generation Sequencing. *Journal of visualized experiments : JoVE* (126).

2. Robinson JT*, et al.* (2011) Integrative genomics viewer. *Nature biotechnology* 29(1):24-26.

3. Roth A*, et al.* (2014) PyClone: statistical inference of clonal population structure in cancer. *Nature methods* 11(4):396-398.

4. Lawrence MS*, et al.* (2013) Mutational heterogeneity in cancer and the search for new cancer-associated genes. *Nature* 499(7457):214-218.

5. Zhou Y*, et al.* (2019) Metascape provides a biologist-oriented resource for the analysis of systems-level datasets. *Nature communications* 10(1):1523.

6. Alexandrov LB*, et al.* (2013) Signatures of mutational processes in human cancer. *Nature* 500(7463):415-421.

7. Mroz EA & Rocco JW (2013) MATH, a novel measure of intratumor genetic heterogeneity, is high in poor-outcome classes of head and neck squamous cell carcinoma. *Oral oncology* 49(3):211-215.

**Supplementary Figure:**

**Supplementary Figure 1 Sequencing depth statistics.** Sequencing depth of 169 tumor samples and paired adjacent samples; The green bar shows the tumor sample, which is based on the left axis; the red dotted line shows the adjacent sample, which is based on the right axis.

**Supplementary Figure 2 Comparative analysis of high-frequency mutation genes in different datasets.** (A) High-frequency mutation genes landscape display of in-house cohort and public datasets; TMB comparison of samples with or without MUC16 (B) and TTN (C) gene mutation in the in-house cohort; (D)Co-occurrence analysis of mutations in MUC16 and TTN genes in the in-house cohort. (E)TMB comparison of samples with or without MUC16 and TTN gene mutation in the public datasets.

**Supplementary Figure 3 The prognostic analysis of extended Laurent classification.** Overall survival of the four histological phenotypes.

**Supplementary Figure 4** **Association analysis of *APC* gene mutation with lauren types.** (A) The proportion of *APC* gene mutations in the four types of in-house cohort; (B) Proportion of *APC* mutations in instestinal type and diffuse type in different datasets (C) Proportion of MSI value with and without *APC* mutation in different datasets;(D)The effect of *APC* mutation on prognosis in different datasets.

**Supplementary Figure 5** **Distribution and prognostic effect of CA19-9 and CEA in patients.** Prognostic analysis of CA19-9 (A) and CEA (B) in cohort; The distribution of CA19-9 (C) and CEA (D) in the four types; Prognostic analysis of CA19-9 (E) and CEA (F) in NSRD group.

**Supplementary Figure 6 Base substitution pattern and variation type statistics.** (A, C, E, G) The bar graph shows the 96-base substitution patterns of the four types. (B, D, F, H) Gene variation types, classification and base substitution statistics in four types.

**Supplementary Table:**

Supplementary Table 1 Statistics of clinical indicators

**
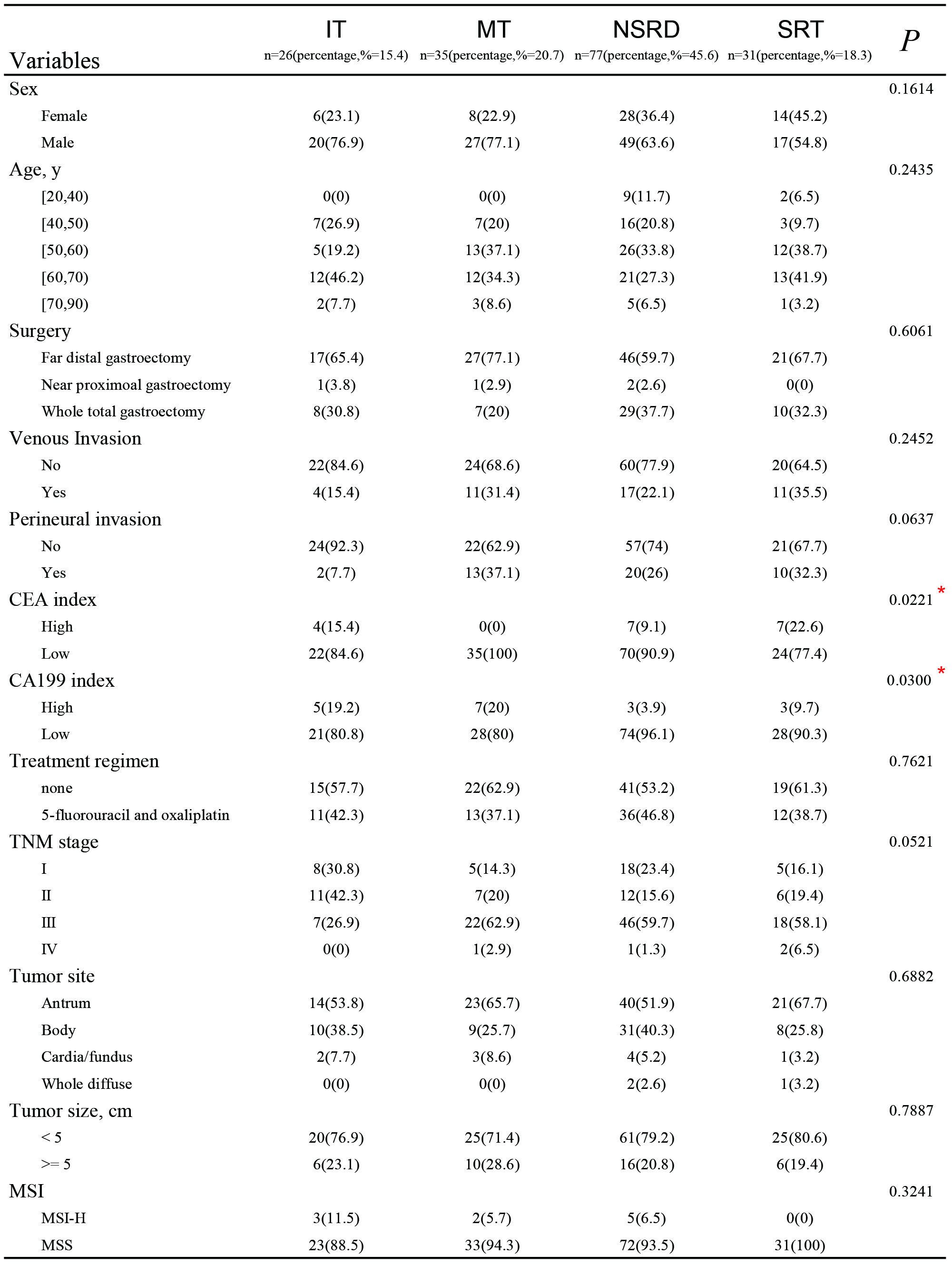
**

The red asterisk represent the p value less than 0.05 with Chi-square test.

**Supplementary Table 2 This file contains five Tables** Table 1 Clinical metadata of 169 cases in our cohort. Table 2 Mutations identified within the panel data. Table 3 clone number and prognostic data. Table 4 Recurrent mutations gene in the 169 cases by MutSigCV. Table 5 panel gene list.
